# A Novel Radiation Shield for Interventional Echocardiographers With Application During Structural Heart Disease Procedures

**DOI:** 10.1016/j.jacasi.2025.07.021

**Published:** 2025-09-13

**Authors:** Akihisa Kataoka, Takeshi Takata, Ayaka Yanagawa, Kento Kito, Taiga Katayama, Hideyuki Kawashima, Takeyuki Sajima, Yuko Toda, Kunihiro Sakoda, Yusuke Watanabe, Ken Kozuma, Hodaka Nakanishi, Jun’ichi Kotoku

**Affiliations:** a Division of Cardiology, Department of Internal Medicine, Teikyo University, Tokyo, Japan; b Advanced Comprehensive Research Organization, Teikyo University, Tokyo, Japan; c Graduate School of Medical Care and Technology, Teikyo University, Tokyo, Japan; d Department of Anesthesia, Teikyo University, Tokyo, Japan; e Department of Cardiology, Tokyo Medical University Hachioji Medical Center, Tokyo, Japan; f MS Group Co, Ltd, Kiyosu, Aichi, Japan

**Keywords:** interventional echocardiographer, radiation exposure, structural heart disease, transesophageal echocardiography, workability

## Abstract

**Background:**

During structural heart disease procedures, interventional echocardiographers are exposed to high doses of radiation, particularly in their right waist and lower body regions. Currently, no radiation shields that are practical for use during transesophageal echocardiography are available.

**Objectives:**

The authors aimed to develop a novel radiation shield that enables interventional echocardiographers to perform transesophageal echocardiography with minimal radiation exposure during structural heart disease procedures.

**Methods:**

A shape prototype model, including a freestanding radioprotective board with lateral windows, was used to assess clinical usability and Monte Carlo simulations were employed. Real-life radiation exposures, both with and without completed shields, were measured during 193 consecutive structural heart disease procedures (114 transcatheter edge-to-edge repairs of the mitral valve and 79 transcatheter aortic valve replacements).

**Results:**

The shape prototype experiment determined the optimal window size to be 300 mm wide and 200 mm high. The actual use of the shield was trouble-free in all procedures. Real-life exposure measurements showed a significant reduction in both types of procedures when the shield was used, especially around the waist (median: 0-8.0 μSv; *P* < 0.001 for transcatheter edge-to-edge repair and median: 0-14.0 μSv; *P* < 0.001 for transcatheter aortic valve replacement).

**Conclusions:**

Through assessment of clinical usability using a shape prototype model and Monte Carlo simulations, a novel radiation shield with practical use was developed for interventional echocardiographers. Clinical studies and simulations demonstrated that this shield is practical for transesophageal echocardiography monitoring and guidance while providing sufficient radiation protection during structural heart disease procedures. (Japanese Development of radiation protection plates for catheter surgery for structural heart disease [echocardiologist and anesthesiologist]; UMIN000046478)

During a structural heart disease (SHD) procedure, interventional echocardiographers performing transesophageal echocardiography (TEE) in cardiac catheterization or hybrid cardiac surgical suites are exposed to higher doses of radiation than the operator of the first catheter.^1^, ^2^, ^3^ We previously reported that the right half of the interventional echocardiographer’s body, especially the waist and lower body, was exposed to high levels of radiation when no protective devices were used.^4^^,^^5^ This suggests that education and appropriate shielding regarding radiation protection during SHD procedures are warranted for interventional echocardiographers, especially young female doctors just starting their careers, as this career phase often coincides with their childbearing years.^6^^,^^7^

Appropriate use of protective equipment—such as mobile ceiling-mounted lead screens and lead curtains, which dramatically reduce exposure for interventional echocardiographers—is recommended.^1^^,^^8^, ^9^, ^10^ However, the equipment is typically only used by catheter operators in cardiac catheterization suites. It is not readily available to interventional echocardiographers in hybrid cardiac surgical suites because of ceiling-mounted C-arms, shadowless lights, high-efficiency particulate air filters, and surgical beds based on product specifications.^11^^,^^12^ Therefore, hybrid cardiac surgical suites may opt to utilize a freestanding floor-mounted protective board as a solution. However, this approach may pose challenges, particularly regarding interference with TEE equipment and probe handling. In clinical practice, facilities are adopting various measures, including utilizing mobile height-adjustable accessory shields up to waist height or combining 2 large leaded acrylic shields.^3^^,^^10^ A prototype protection device for interventional echocardiographers and anesthetists during SHD procedures has also been reported.^13^ However, no devices that enable interventional echocardiographers to perform TEE monitoring and guidance (workability) are currently available. Therefore, in this study, we aimed to develop a radiation shield resembling a large floor-mounted protective board with a novel design with workability while minimizing radiation exposure (shielding) during SHD procedures. To achieve this, we assessed clinical usability using a shape prototype model and Monte Carlo simulations.

## Methods

### Consent

The study protocol was developed following the 1975 Declaration of Helsinki and its later amendments, and was approved by the Institutional Review Board of Teikyo University (approval number, TEIRIN 18-176, 20-178, and 21-100). All participants provided written informed consent. This trial was registered with the University Hospital Medical Information Network (UMIN000046478).

### Monte Carlo simulation of radiation exposure of interventional echocardiographers

The Monte Carlo method was employed to simulate radiation exposure in the diagnostic energy range using an in-house software known as the fast dose estimation system for interventional radiology.^4^^,^^9^^,^^11^^,^^14^, ^15^, ^16^, ^17^, ^18^ This system simulates the transport of each photon based on probabilities of photon interactions, thereby providing physically accurate radiation exposure dose calculations. More details are provided in the Supplemental Methods.

**Figure 1:**
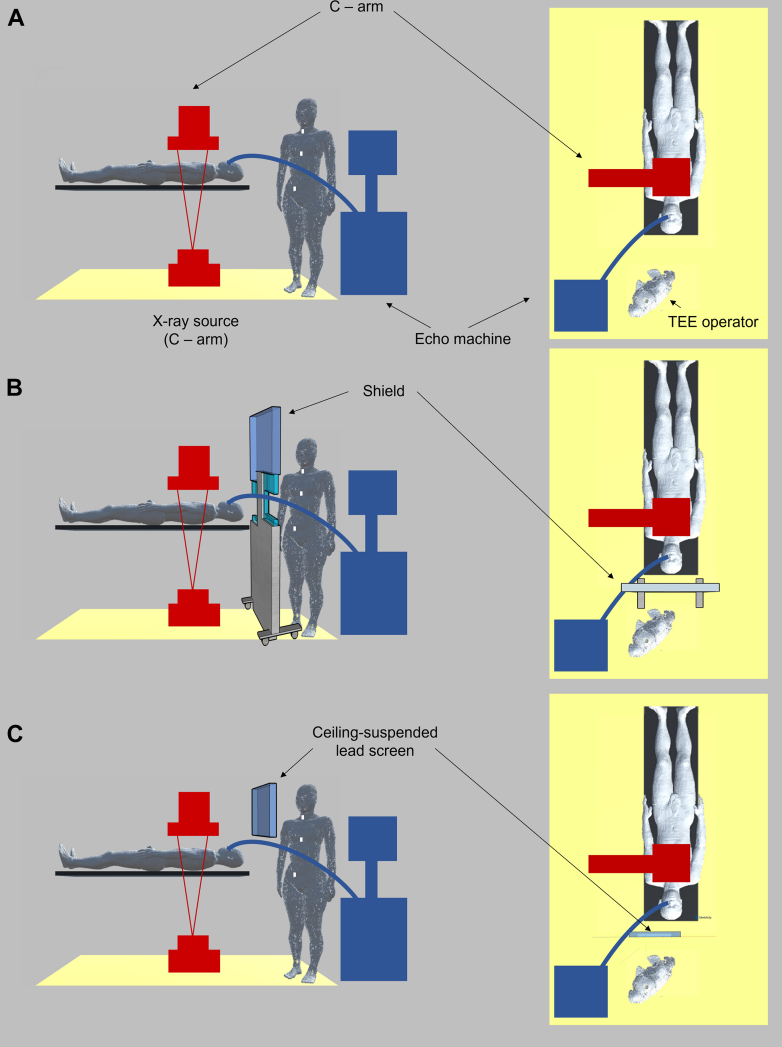
Clinical and Simulation Setup in Hybrid Cardiac Surgery Suite The C-arm and transesophageal echocardiography (TEE) machines were excluded from the simulation. Simulation models: (A) without a shield, (B) with a shield, and (C) with a ceiling-mounted lead screen are shown. In the simulation, the vertical distance from the x-ray source to the shield (ceiling-mounted lead screen) and the airline distances from the center of the x-ray source to the body surface of the interventional echocardiographer were set to 670 and 882 mm, respectively. The ceiling-mounted lead screen was positioned with its upper edge at a height of 160 cm from the floor, near the head.

Figure 1A depicts the cardiac surgery suite setup at our institution, including an under-table x-ray source, bed, air, concrete floor, a whole-body model of a male patient, and a whole-body model of a female interventional echocardiographer performing TEE during an SHD procedure. This was set to the average height of Japanese individuals (160.8 cm).^19^ C-arm and echo machines were not included in the simulation. Figures 1B and 1C show the installation of the 1-mm Pb shield and 1-mm Pb ceiling-mounted screen (height 40 mm, and width 50 mm, based on the most commonly used size in Japanese catheter laboratories). In the simulation, the vertical distance from the x-ray source to the shield (ceiling-mounted lead screen) and the straight line distance from the center of the x-ray source to the surface of the interventional echocardiographer’s body were set to 670 and 882 mm, respectively. These distances were measured and set based on the actual distances of transcatheter edge-to-edge repair (TEER) on a certain day. The ceiling-mounted lead screen was positioned with its upper edge at a height of 160 cm from the floor (near the head).

### Experiments for the optimal size of the window

**Figure 2:**
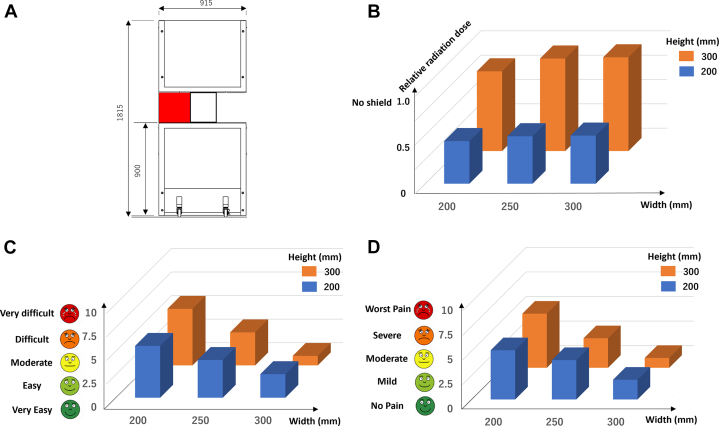
Experiments for Shield Design Using a Shape Prototype Model (A) Design blueprint of the shape prototype model, wherein the size of the left window (highlighted in red) is altered. (B) Monte Carlo simulations to assess the relative radiation exposure of the surface of the right waist based on the window size, assuming a cusp overlap view. A relative radiation dose of 1.0 represented the dose without shield protection. (C) The ease of transesophageal echocardiography probe handling (0 to 10: very easy to very difficult). (D) The physical burden experienced by the interventional echocardiographer during transesophageal echocardiography (0 to 10: no pain to worst pain) based on the window size.

Following the design of a typical hybrid surgery suite, a shape prototype was created to accommodate a freestanding radioprotective board (height 1,815 mm; width 915 mm) (Figure 2A). The board featured cutout windows on both sides to facilitate TEE and anesthesia. The size of the lateral windows (Figure 2A) directly affected workability; larger openings ensured ease of operation but simultaneously increased radiation exposure through these apertures.

We assessed the ease of TEE probe handling and the physical burden on the interventional echocardiographer using a self-reported scale ranging from 0 (very easy) to 10 (very difficult) and from 0 (no pain) to 10 (worst pain) for each window size. The size of the window was defined by the lateral (width) and vertical (height) dimensions, using a phantom patient model placed on the patient’s bed to determine the optimal window design considering workability. For the shielding analysis, we used the previously mentioned Monte Carlo simulations to compute the dose of radiation to which the body surface of the interventional echocardiographer’s right waist, which typically receives the highest radiation exposure, was exposed. This was done for each window size, assuming a transcatheter aortic valve replacement (TAVR) procedure with a C-arm angle of cusp overlap view (right anterior oblique 9°–caudal 1°), during which interventional echocardiographers are exposed to high doses of radiation.^4^

### Clinical study for efficacy of the shield

This prospective, nonrandomized, single-center study included 229 consecutive SHD procedures using continuous TEE. The radiation shield was introduced on February 14, 2022, and used in all subsequent procedures (shield group) (Central Illustration A, Figure 1B), whereas in cases treated before this date, the procedure was performed without the shield (control group) (Figure 1A).

**Central Illustration:**
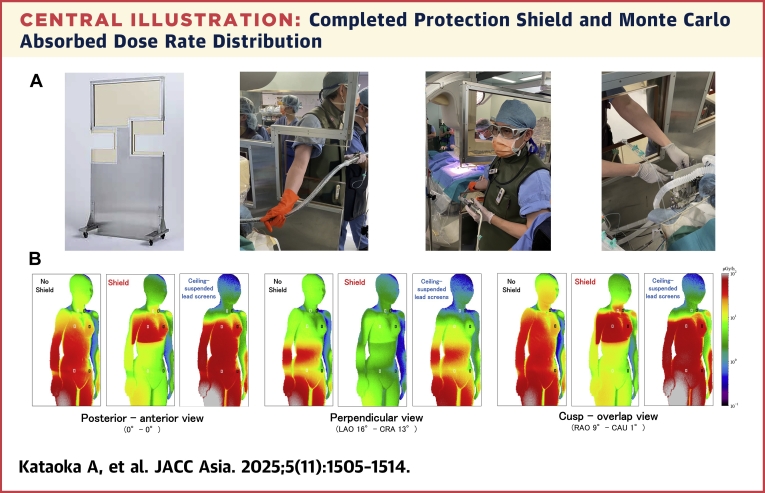
Completed Protection Shield and Monte Carlo Absorbed Dose Rate Distribution (A) The shield provides complete protection from the x-ray source (left). The 3, 1-mm Pb transmitting acrylic panels (light yellow) and the 4 casters are included. The figure depicts its actual use in transcatheter edge-to-edge repair procedures, showing the x-ray source, patient’s bed (middle left), and interventional echocardiographers (middle right). An additional window for the anesthesiologist is located on the opposite side (right). (B) The absorbed dose rate distributions in the body surface of the interventional echocardiographer in the posterior–anterior, perpendicular, and cusp overlap views. The left panels represent front views of an interventional echocardiographer model without any shield (equivalent to the control group in the clinical study). The middle and right panels represent front views with the shield (equivalent to the shield group in the clinical study) and with a ceiling-mounted lead screen, respectively. Areas that receive high doses of radiation (>20 Gy/h) are shown in red. CAU = caudal; CRA = cranial; LAO = left anterior oblique; RAO = right anterior oblique.

**Figure 3:**
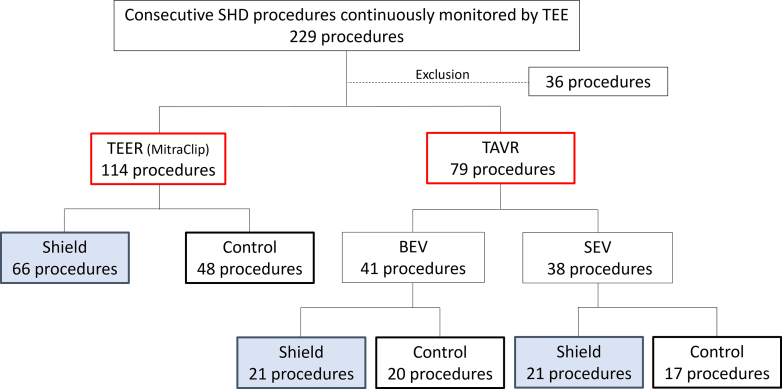
Clinical Study Flowchart Before February 14, 2022, no external shields or absorbing devices were used in any of the 85 procedures (control group). After February 14, 2022, the shield was used in all 108 procedures (shield group). BEV = balloon-expandable valve; SEV = self-expanding valve; SHD = structural heart disease; TAVR = transcatheter aortic valve replacement; TEE = transesophageal echocardiography; TEER = transcatheter edge-to-edge repair.

Of these, 36 procedures, comprising TEER for the tricuspid valve (n = 1), left atrial appendage closure (n = 27), and patent foramen ovale closure (n = 8), were excluded because of the limited sample size in control procedures. Therefore, 114 TEERs of the mitral valve using the MitraClip device (Abbott Vascular), and 79 TAVRs conducted between April 1, 2021, and October 31, 2023 (Figure 3) were included for the final analysis in the study. All procedures were performed by 2 or 3 interventional attending cardiologists (each with >15 years of experience) and 4 echocardiography fellows (each with >5 years of experience) under the consistent guidance and supervision of an echocardiography-attending cardiologist with 20 years of experience in clinical practice.

A semiconductor personal radiation dosimeter (Hitachi) was used to measure the radiation dose at the surface of the interventional echocardiographer’s neck (right side), chest (left pocket of the radiation-protective clothing), and waist (right side). As described in our previous report, the dosimeters were attached to the outside of the radiation-protective clothing.^4^ In addition, fluoroscopy and procedure time, and cumulative air kerma, which was defined as the total radiation energy delivered to a specific reference point in air throughout the entire procedure, were obtained during each procedure using a fluoroscopic system (Allura Xper FD20, Philips Medical Systems). Initially, the experimental setup was designed to represent the typical setup in our institution’s hybrid cardiac surgery suite. Therefore, no external shields, such as free-standing shields, ceiling-mounted lead screens, or absorbing devices (RADPAD, Worldwide Innovations & Technologies), were used in this study (control group) (Figure 1A).

### Statistical analysis

Continuous variables are presented as median (IQR), whereas categorical variables are presented as number (percentage). Given the relatively small sample size, we used the non-parametric Mann-Whitney *U* test to compare continuous variables. The chi-square test was used to compare categorical variables expressed as numbers and percentages. All statistical analyses were performed using MedCalc software version 20.2 (MedCalc Software). Statistical significance was set at *P* < 0.05.

## Results

### Experiments for the design of the shield using a shape prototype model

Ten echocardiologists (4 attending and 6 fellows; 6 men; median height: 171 [Q1-Q3: 155.0-177.0] cm) who could perform TEE examination participated in the experiments. Monte Carlo simulations demonstrated that as the window width or height increased, so did the radiation dose to the surface of the waist. A notable increase in the dose was observed when the height increased from 200 to 300 mm (Figure 2B). Moreover, the difficulty in handling the TEE probe and the physical burden placed on the interventional echocardiographers increased as the width or height decreased (Figures 2C and 2D). However, the impact of the decreased height was smaller than that of the decreased width under both conditions. The grid search of these results suggested that the optimal window size should be a width of 300 mm and a height of 200 mm to ensure workability and achieve minimal radiation exposure without imposing a physical burden on the TEE operator.

### Shield and Monte Carlo simulation

Based on the design of the window for the TEE operator determined in the shape prototype experiment, we commissioned the MS Group Co., Ltd. (Kiyosu, Aichi, Japan) to create the radiation shield. This was successfully completed (Central Illustration A). The dimensions of the shield are 1,815 mm in height, 915 mm in width, and 500 mm in depth (at the legs); it weighs 65 kg and features 4 casters, facilitating easy movement. This innovative radiation shield comprises 3, 1-mm Pb transmitting acrylic panels that can be detached using a cassette mechanism. One panel is positioned at the top, and the remaining 2, which are equipped with windows of optimal size, are positioned on both sides with a lead-lined iron plate to protect against x-rays. It offers 2 different window heights from the surface of the floor that can be adjusted by swapping the top and bottom of the panel to accommodate the height of the TEE operator. The TEE probe can be maneuvered through the window (Central Illustration A). The rate distributions of the dose absorbed by the interventional echocardiographer’s body surface, evaluated using Monte Carlo simulations (Central Illustration B), showed that the protective shield reduced radiation exposure in all views (Central Illustration) compared with a setup without the shield (Central Illustration). However, in the posterior-anterior and cusp overlap views, a high-dose region continued to appear in the right chest. The radiation dose to the head and neck was reduced with the use of the ceiling-mounted lead screen (Central Illustration) compared with the setup without the shield; however, part of the chest and the area below it remained exposed to high levels of radiation.

### Clinical study for efficacy of the shield

In total, 108 of the 193 procedures used only a shield without any other external shields (shield group), whereas 85 procedures did not use any radiation shields (control group) (Figure 3). Using the complete shield posed no issues for handling the TEE probe, and no technical difficulties were encountered during the procedure. Even with a steeper caudal view, where the x-ray source is moved closer to the shield, the ease of movement reduced the risk of accidental contact.

The procedural and radiation exposure data for the TEER procedures are shown in Table 1. No significant differences in cumulative air kerma between the 2 groups were observed. Greater reductions in radiation exposure were found at all positions in the shield group (neck, median: 1.0 vs 5.5 μSv; *P* < 0.001; chest, median: 0 vs 1.0 μSv; *P* < 0.001; waist, median: 0 vs 10.0 μSv; *P* < 0.001) (Table 1).

**Table 1:** Patient Characteristics and Procedural and Radiation Dose Data in TEER Procedures

	Shield Group (n = 66)	Control Group (n = 48)	*P* Value
Male,	40 (60.6)	23 (47.9)	0.180
Age, y	80.5 (73.0-86.0)	80.0 (71.5-85.0)	0.689
Body mass index, kg/m^2^	19.9 (17.5-22.2)	19.0 (17.4-22.0)	0.618
Hypertension	45 (68.1)	28 (58.3)	0.281
Dyslipidemia	29 (43.9)	16 (33.3)	0.255
Diabetes mellitus	21 (31.8)	11 (22.9)	0.299
Ever-smokers	15 (22.7)	12 (25.0)	0.779
Chronic kidney disease	61 (92.4)	43 (89.5)	0.598
Atrial fibrillation	38 (57.5)	30 (62.5)	0.598
Peripheral artery disease	6 (9.1)	1 (2.1)	0.126
Coronary artery disease	16 (24.2)	16 (33.3)	0.288
Previous stroke	0 (0.0)	8 (16.7)	<0.001
Chronic obstructive pulmonary disease	0 (0.0)	2 (4.2)	0.096
Fluoroscopy time, min	11.4 (9.5-13.9)	15.5 (11.0-22.9)	<0.001
Procedure time, min	41.0 (35.0-47.0)	43.0 (31.0-60.5)	0.514
CAK, mGy	25.3 (17.8-33.4)	26.2 (17.3-58.7)	0.131
Neck, μSv	1.0 (0.0-2.0)	5.5 (3.0-9.0)	<0.001
Chest, μSv	0 (0-0)	1.0 (0-3.5)	<0.001
Waist, μSv	0 (0-0)	10.0 (5.5-15.0)	<0.001

Values are n (%) or median (IQR).

Chronic kidney disease was defined as estimated glomerular filtration rate <60 mL/min/1.73 m^2^.

CAK = cumulative air kerma; TEER = transcatheter edge-to-edge repair.

During the study, a total of 79 TAVR procedures were conducted, 41 of which used balloon-expandable valves (SAPIEN 3 series, Edwards Lifesciences) and 38 of which used self-expanding valves (35 Evolut family, Medtronic, and 3 Navitor, Abbott) (Figure 3, Table 2). Notably, the shield group underwent a substantially higher number of transfemoral approach procedures, both overall and in the balloon-expandable valve subgroups. Across the entire cohort, we observed significant reductions in radiation exposure across all positions in the shield group when compared with the control group. Specifically, in the balloon-expandable valve subgroup analysis, the shield group showed substantially lower radiation doses than the control group at all locations. However, in the self-expanding valve subgroup analysis, the shield group only demonstrated a significant reduction in radiation dose at the waist when compared with the control group (Table 2).

**Table 2:** Patients’ Characteristics and Procedural and Radiation Dose Data in TAVR Procedures

	Overall (N = 79)			BEV (N = 41)			SEV (N = 38)		
	Shield Group (n = 42)	Control Group (n = 37)	*P* Value	Shield Group (n = 21)	Control Group (n = 20)	*P* Value	Shield Group (n = 21)	Control Group (n = 17)	*P* Value
Age, y	85.0 (80.0-89.0)	84.0 (79.0-87.0)	0.296	85.0 (78.0-89.0)	80.5 (77.5-85.5)	0.301	86.0 (81.7-88.2)	84.0 (82.5-88.2)	0.836
Male	17 (40.4)	13 (35.1)	0.627	9 (42.8)	8 (40.0)	0.854	8 (38.0)	5 (29.4)	0.579
Body mass index, kg/m^2^	20.8 (19.0-22.8)	21.7 (19.0-24.3)	0.539	20.9 (19.8-23.3)	20.9 (18.8-25.3)	0.916	20.7 (17.8-22.7)	21.8 (19.1-23.3)	0.522
Hypertension	24 (57.1)	13 (73.0)	0.145	13 (61.9)	16 (80.0)	0.209	11 (52.4)	11 (64.7)	0.450
Dyslipidemia	17 (40.5)	13 (35.1)	0.628	7 (33.3)	5 (25.0)	0.563	10 (47.6)	8 (47.1)	0.972
Diabetes mellitus	7 (16.7)	11 (29.7)	0.170	5 (23.8)	5 (25.0)	0.930	2 (9.5)	6 (35.3)	0.056
Ever-smokers	4 (9.5)	7 (18.9)	0.232	0 (0.0)	5 (25.0)	0.016	4 (19.0)	2 (11.8)	0.546
Chronic kidney disease	31 (73.8)	30 (81.1)	0.445	15 (71.4)	17 (85.0)	0.300	16 (76.2)	13 (76.5)	0.984
Atrial fibrillation	14 (33.3)	12 (32.4)	0.933	8 (38.1)	6 (30.0)	0.589	6 (28.6)	6 (35.3)	0.662
Peripheral artery disease	5 (11.9)	4 (10.8)	0.879	3 (14.3)	1 (5.0)	0.323	2 (9.5)	3 (17.6)	0.467
Coronary artery disease	10 (23.8)	14 (37.8)	0.179	5 (23.8)	5 (25.0)	0.930	5 (23.8)	9 (52.9)	0.068
Previous stroke	2 (4.8)	6 (16.2)	0.094	1 (4.8)	2 (10.0)	0.525	1 (4.8)	4 (23.5)	0.093
Chronic obstructive pulmonary disease	1 (2.4)	1 (2.7)	0.928	0 (0.0)	1 (5.0)	0.306	1 (4,8)	0 (0.0)	0.368
Approach			0.013			0.016			0.281
Transfemoral	23 (54.8)	30 (81.1)		9 (42.9)	16 (80.0)		14 (66.7)	14 (82.4)	
SCA	19 (45.2)	7 (17.9)		12 (57.1)	4 (20.0)		7 (33.3)	3 (17.6)	
Fluoroscopy time, min	13.1 (11.2-16.7)	15.4 (11.7-17.1)	0.389	11.2 (9.1-12.5)	14.0 (8.6-16.1)	0.210	16.5 (14.4-20.0)	16.6 (14.7-19.2)	0.929
Procedure time, min	45.5 (40.0-53.0)	44.0 (35.5-56.0)	0.360	43.0 (38.0-55.0)	40.5 (33.5-49.5)	0.403	47.0 (39.7-54.7)	45.0 (38.0-56.5)	0.735
CAK, mGy	286.7 (205.3-424.3)	274.2 (213.1-375.1)	0.926	219.2 (152.8-283.3)	279.6 (211.0-388.3)	0.065	401.0 (301.8-492.0)	253.0 (214.3-349.3)	0.597
Neck, μSv	5.5 (2.0-10.0)	11.0 (4.0-16.2)	0.009	4.0 (0.7-7.0)	9.5 (4.0-16.0)	0.004	9.0 (2.7-13.2)	11.0 (4.0-17.0)	0.377
Chest, μSv	1.0 (0.0-5.0)	3.0 (1.0-9.2)	0.006	0 (0.0-3.0)	4.5 (1.5-9.0)	0.006	1.0 (0.0-8.2)	1.2 (1.0-14.5)	0.209
Waist, μSv	0 (0-0)	14.0 (7.7-43.0)	<0.001	0 (0-0)	11.5 (6.5-33.0)	<0.001	0 (0-1.0)	17.0 (10.0-47.7)	<0.001

Values are median (IQR) or n (%).

Chronic kidney disease was defined as estimated glomerular filtration rate <60 mL/min/1.73 m^2^.

BEV = balloon-expandable valve; CAK = cumulative air kerma; SCA = subclavian artery; SEV = self-expanding valve; TAVR = transcatheter aortic valve replacement.

## Discussion

To our knowledge, this is the first study in which a commercially available radiation shield to protect interventional echocardiographers during SHD procedures that considers both workability and shielding ability was developed in Japan. The study was designed to assess clinical usability using a shape prototype model and Monte Carlo simulation. The clinical study results were consistent with those of the simulation and showed a remarkable reduction in radiation exposure, especially to the waist and lower body. The main advantage of this shield lies in its ability to preserve TEE workability; during its actual use, no issues were encountered with handling the TEE. Furthermore, the shield did not affect the positional relationship between the echocardiography machine and the TEE probe, and therefore did not affect image quality (Supplemental Figures 1 and 2). This was reflected in the results, with no significant difference being observed in procedure time between the shield and control groups for the TEER and TAVR procedures.

In the United States and Japan, more female doctors practice echocardiography than other invasive subspecialties.^7^^,^^20^ Radiation exposure to the abdominal surface and to the embryo or fetus in pregnant health care workers should not exceed 2 and 1 mSv, respectively, according to the recommendations of the International Commission on Radiological Protection and U.S. federal law, respectively.^11^^,^^12^^,^^21^^,^^22^ Radiation exposure for interventional echocardiographers is mainly derived from radiation scattering at the bottom edge of the patient bed.^4^ Therefore, using a ceiling-mounted lead screen leads to a minimal reduction in radiation exposure below the chest. In particular, this results in high radiation exposure to the lower body surface. However, in the Monte Carlo simulation, the shield significantly reduced radiation exposure to the lower abdomen and legs. Consequently, the real-life measurements revealed significantly higher exposure of the body surface to radiation when no protection was provided, particularly in the waist and lower regions where the ovaries are located.^4^^,^^23^ Recent discussions highlight concerns regarding fetal radiation exposure in female interventional echocardiographers.^24^ Therefore, recognizing that female doctors at childbearing age are particularly at risk of exposure to high doses of radiation and require appropriate shielding measures is imperative.

Our innovative shield reduced the real-life radiation dose in the waist region to almost zero, making it highly effective. Nevertheless, scattered radiation still passed through the window portion of the shield. As previously mentioned, a tradeoff between workability and shielding exists, and this balance has been optimized in this model. Monte Carlo simulations showed high radiation exposure in the right chest and neck during certain views, but actual measurements confirmed significant dose reductions during both TEER and TAVR, especially with balloon-expandable valves. By contrast, no significant reduction was observed in the neck and chest with self-expanding valves, likely due to the frequent use of the cusp-overlap view.^25^ Thus, we recommend continued use of protective clothing and neck guards in addition to the shield. Moreover, positioning the torso closer to the center of the shield center may further reduce exposure to scattered radiation. Furthermore, the right hand of the TEE operator, used to manipulate the TEE probe, extends beyond the shield towards the x-ray source, making exposure of the hand skin to radiation inevitable. Therefore, exercising caution by avoiding unnecessary extension of the hand toward the x-ray equipment is essential. Additional precautions, such as wearing lead-lined gloves and a humeral shield, are also necessary.

SHD procedures are typically performed under general or local anesthesia.^26^ As more young women become anesthesiologists,^27^ protecting them from radiation is particularly important. Although this study focused on shielding for interventional echocardiologists, the shield also includes a side window for anesthesiologists (Central Illustration A). We previously demonstrated its effectiveness through a Monte Carlo simulation.^18^ Covering the echocardiologist’s side window with lead-lined plates further reduces exposure during other procedures such as aortic stent graft or endovascular neurosurgery. Clinical data on radiation exposure in anesthesiologist will be reported in a future study.

### Study Limitations

First, interventional echocardiographers wore radiation-protective clothing, which was not considered in the Monte Carlo simulations. In addition, dosimeters were attached to the outside surface of the protective clothing. Therefore, the clinical study may have drastically overestimated the radiation dose to which the body surface of the interventional echocardiographer was exposed. Second, the neck and chest (including the breasts) remain exposed to high doses of radiation with the current shield design, especially when the cusp overlap view of TAVR is conducted. Third, it may not be common for interventional echocardiographers to perform TEE monitoring during TAVR cases at many institutions because of spreading local anesthesia TAVR. Fourth, the radiation dose rate tends to be proportional to both the tube current and frame rate. This relationship arises from increases in tube current and frame rates due to factors such as the patient’s physique. Consequently, a high body mass index in the patient emerged as the most significant predictor of radiation exposure among operators conducting diagnostic cardiac angiography.^28^ This clinical study was performed in Japan, where patients typically have a lower body mass index compared with those in Western countries.^29^ As a result, radiation exposure doses may vary between these populations. Furthermore, the echocardiologists who participated in the shape prototype experiment were believed to be of smaller stature than the average in Western countries; therefore, the shield may not be as effective for non-Japanese individuals. Fifth, this was a single-center observational study and, in part, a simulation study using the cardiac surgery hybrid suite setup from our institution. The scattering effects may vary depending on the specific placement of the equipment, which differs among institutions. Our cardiac surgery hybrid suite has a ceiling-mounted single C-arm x-ray system; however, in some facilities, a floor-mounted C-arm x-ray system or biplane fluoroscopy system is used. In such facilities, the C-arm system may obstruct the workspace of the interventional echocardiographer performing the TEE, making the use of the shield challenging. Furthermore, because a ceiling-mounted lead screen was not installed in our hybrid cardiac surgery suite, it was not used in either the control or shield groups in the clinical study. Additionally, the ceiling-mounted lead screen used in the Monte Carlo simulation may differ in size from those used in countries and regions other than Japan. Sixth, we did not perform a formal sample size calculation prior to study initiation; however, all eligible consecutive cases within the defined study period were included. Post hoc power analyses based on the observed effect sizes—such as the significant reduction in waist-level radiation exposure—confirmed that the study was sufficiently powered to detect clinically meaningful differences. Finally, the TEER for the tricuspid valve, left atrial appendage closure, and patent foramen ovale closure, which are also considered SHD procedures, were not included in this study because of the limited sample size. However, no specific problems regarding the workability of the shields used in these procedures have been reported.

## Conclusions

A commercially available novel radiation shield for interventional echocardiographers performing TEE monitoring and guidance during SHD procedures was developed by the assessment of clinical usability using a shape prototype model and Monte Carlo simulation. This allows interventional echocardiographers to maintain TEE workability and enjoy a remarkable degree of protection against radiation exposure. When existing personal protective equipment is supplemented by this shield, safety can be improved during SHD procedures compared to when working without the shield.

### Data Sharing Statement

The data, analytic methods, and study materials will be made available to other researchers for the purposes of reproducing these results or replicating these procedures from the corresponding author upon reasonable request.

## Funding Support and Author Disclosures

This work was supported by the Terumo Life Science Foundation (Kanagawa, Japan), Japan 2020 R&D subsidies, Abbott Medical Japan LLC (Tokyo, Japan) scholarship fund, and the Boston Scientific Co. (Tokyo, Japan) scholarship fund. The Japan Society for the Promotion of Science (grant numbers, 21K07656, 22H05108, and 22K18217) and the Japan Science and Technology Agency ERATO Grant (grant number, JPMJER2102) also supported this work. Financial support was provided to fund the semiconductor personal radiation dosimeters used in this study and the Monte Carlo calculations. Dr Kataoka has received remuneration as a proctor from Abbott Medical Japan. Drs Kataoka, Takata, Yanagawa, and Kotoku have a pending patent in Japan, titled “RADIATION BLOCKING PLATE” (Patent Application No. WO2022/131027) and have licensed this patent to the MS Group Co, Ltd, where Dr Sakoda is the chief operating officer and receives income from it. All other authors have reported that they have no relationships relevant to the contents of this paper to disclose.
