# Supplementary material for: A Novel Radiation Shield for Interventional Echocardiographers With Application During Structural Heart Disease Procedures
Source: JACC Asia. 2025 Sep 13;5(11):1505–14. doi: 10.1016/j.jacasi.2025.07.021 (PMC12673834; doi:10.1016/j.jacasi.2025.07.021)
Supplement: Supplemental Material [file mmc1.docx]

**SUPPLEMENTAL MATERIAL**

**Supplemental Methods**

*Monte Carlo simulation and the simulation system*

The simulation was conducted using a single Tesla P100 graphical processing unit (GPU; NVIDIA Corp., Santa Clara, CA, USA) on a supercomputing system (SGI Rackable C2112-4GP3/C1102-GP8: Reedbush-L; Silicon Graphics International Corp., Milpitas, CA, USA) and a single NVIDIA A100 GPU (NVIDIA Corp., Santa Clara, CA, USA) on the FUJITSU Supercomputer PRIMEHPC FX1000 and FUJITSU Server PRIMERGY GX2570 (Wisteria/BDEC-01; Fujitsu Ltd., Japan) at the Information Technology Center of the University of Tokyo. This study simulated a trillion incident photons with a 5-keV cut-off energy. The simulation suppressed electron transport, thereby accelerating the calculation. Given that the system does not directly compute the absolute dose directly but rather only calculates the relative dose, a conversion factor was established to translate the relative dose into the absolute dose^1–3^.

**Supplemental Figures**

**
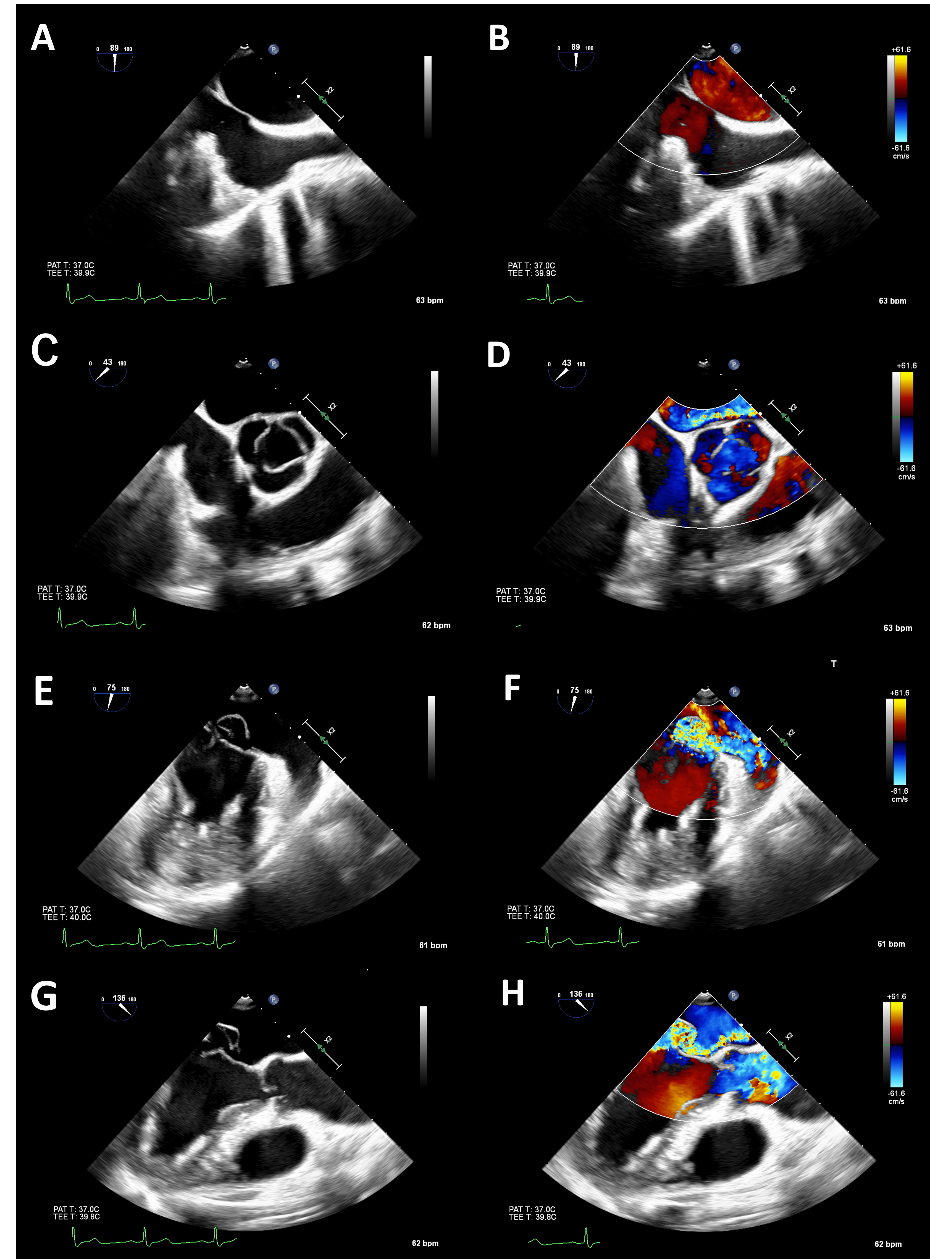
**

**Supplemental Figure 1. TEE images of severe mitral regurgitation due to P2 prolapse with the usage of shield during TEER**

A. Bicaval view image. B. Color-Doppler bicaval view image. C. Short axis view for aortic valve image. D. Color-Doppler short axis view for aortic valve image. E. Inter-commissure view for mitral valve image. F. Color-Doppler inter-commissure view for mitral valve image. G. Left ventricular outflow tract view for mitral valve image. H. Color-Doppler left ventricular outflow tract view for mitral valve image.

Abbreviations: TEE, transesophageal echocardiography; TEER, transcatheter edge-to-edge repair

**
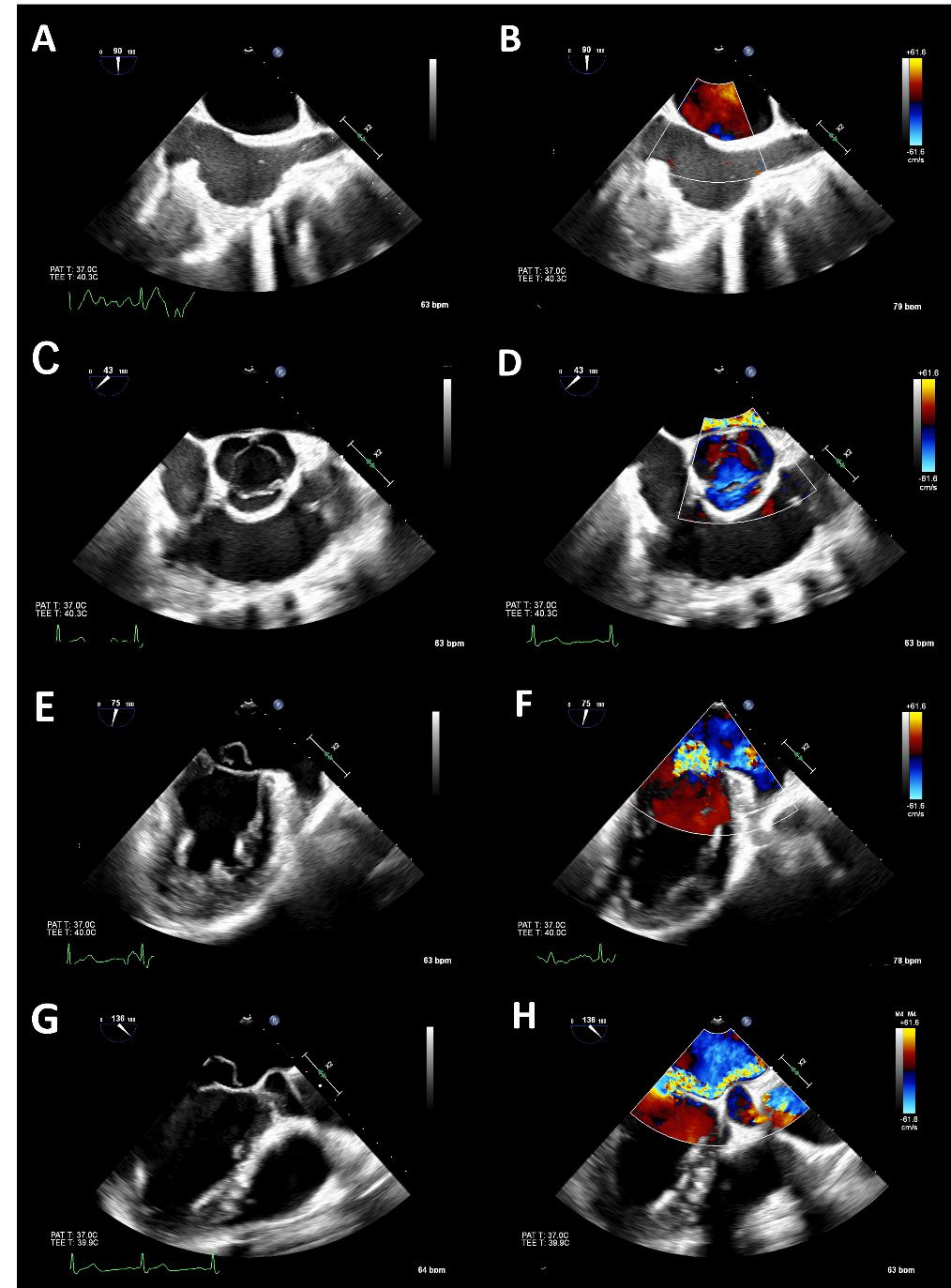
**

**Supplemental Figure 2. TEE images of severe mitral regurgitation due to P2 prolapse without the usage of shield during TEER**

A. Bicaval view image. B. Color-Doppler bicaval view image. C. Short axis view for aortic valve image. D. Color-Doppler short axis view for aortic valve image. E. Inter-commissure view for mitral valve image. F. Color-Doppler inter-commissure view for mitral valve image. G. Left ventricular outflow tract view for mitral valve image. H. Color-Doppler left ventricular outflow tract view for mitral valve image

Abbreviations: TEE, transesophageal echocardiography; TEER, transcatheter edge-to-edge repair

**Supplemental References**

1. Kataoka A, Takata T, Yanagawa A, et al. Body surface radiation exposure in interventional echocardiographers during structural heart disease procedures. JACC: Asia 2023;3:301–309.
2. Takata T, Kataoka A, Yanagawa A, Shiraishi K, Kotoku Ji. Simulation of radiation dose for female physicians: radiation exposure risk in structural heart disease procedures. J Transcatheter Valve Ther 2023;5:17–23.
3. Yanagawa A, Takata T, Onimaru T, et al. New perforated radiation shield for anesthesiologists: Monte Carlo simulation of effects. J Radiat Res 2023;64:379–386.
